# A Resource Planning Analysis of District Hospital Surgical Services in the Democratic Republic of the Congo

**DOI:** 10.9745/GHSP-D-14-00165

**Published:** 2015-03-02

**Authors:** Melanie Sion, Dheepa Rajan, Hyppolite Kalambay, Jean-Pierre Lokonga, Joseph Bulakali, Mathias Mossoko, Dieudonne Kwete, Gerard Schmets, Edward Kelley, Tarcisse Elongo, Luis Sambo, Meena Cherian

**Affiliations:** a Thomas Jefferson University, Department of Surgery, Philadelphia, PA, USA.; b World Health Organization (WHO), Department of Health Systems Governance and Financing, Geneva, Switzerland.; c Ministry of Health, Directorate of Planning, Kinshasa, Democratic Republic of the Congo (DRC).; d WHO Country Office, Kinshasa, DRC.; e Ministry of Health, Global Fund Country Coordination Mechanism Secretariat, Kinshasa, DRC.; f Ministry of Health, Directorate of Primary Health Care Development, Kinshasa, DRC.; g Prime Minister's Office, Kinshasa, DRC.; h WHO, Service Delivery and Safety Department, Geneva, Switzerland.; i WHO AFRO Regional Office, Brazzaville, Republic of Congo.

## Abstract

**Background:**

The impact of surgical conditions on global health, particularly on vulnerable populations, is gaining recognition. However, only 3.5% of the 234.2 million cases per year of major surgery are performed in countries where the world's poorest third reside, such as the Democratic Republic of the Congo (DRC).

**Methods:**

Data on the availability of anesthesia and surgical services were gathered from 12 DRC district hospitals using the World Health Organization's (WHO's) Emergency and Essential Surgical Care Situation Analysis Tool. We complemented these data with an analysis of the costs of surgical services in a Congolese norms-based district hospital as well as in 2 of the 12 hospitals in which we conducted the situational analysis (Demba and Kabare District Hospitals). For the cost analysis, we used WHO's integrated Healthcare Technology Package tool.

**Results:**

Of the 32 surgical interventions surveyed, only 2 of the 12 hospitals provided all essential services. The deficits in procedures varied from no deficits to 17 services that could not be provided, with an average of 7 essential procedures unavailable. Many of the hospitals did not have basic infrastructure such as running water and electricity; 9 of 12 had no or interrupted water and 7 of 12 had no or interrupted electricity. On average, 21% of lifesaving surgical interventions were absent from the facilities, compared with the model normative hospital. According to the normative hospital, all surgical services would cost US$2.17 per inhabitant per year, representing 33.3% of the total patient caseload but only 18.3% of the total district hospital operating budget. At Demba Hospital, the operating budget required for surgical interventions was US$0.08 per inhabitant per year, and at Kabare Hospital, US$0.69 per inhabitant per year.

**Conclusion:**

A significant portion of the health problems addressed at Congolese district hospitals is surgical in nature, but there is a current inability to meet this surgical need. The deficient services and substandard capacity in the surveyed district hospitals are systemic in nature, representing infrastructure, supply, equipment, and human resource constraints. Yet surgical services are affordable and represent a minor portion of the total operating budget. Greater emphasis should be made to appropriately fund district hospitals to meet the need for lifesaving surgical services.

## Abstract (precis)

District hospitals in the DRC, on average, could not provide 21% of lifesaving surgical interventions due to deficiencies in basic infrastructure and essential surgical equipment and supplies. Surgery's important health impact and proportionally low service delivery budget argue for greater emphasis on surgical interventions, including for obstetric care.

## BACKGROUND

The impact of surgical conditions on global health, and particularly on vulnerable populations such as women and children, the poor, and rural communities, is gaining recognition. In fact, Debas and colleagues found that 11% of the worldwide global burden of disease is due to surgical conditions, including injuries, malignancies, congenital anomalies, and obstetrical complications.^1^ However, of the estimated 234.2 million major surgical cases per year performed,^2^ only 3.5% of them are performed in countries where the world's poorest third reside, such as the Democratic Republic of the Congo (DRC).

Only 3.5% of major surgical cases are performed in countries where the world's poorest third reside.

Is surgery only for the rich? Emerging evidence sheds light on misconceptions regarding surgical care delivery, such as its high service resource needs with tertiary-level technical requirements.^3^ Furthermore, studies have shown the critical need for access to quality surgical obstetric care to reduce maternal mortality. Based on data from a number of studies, Ronsmans et al. concluded that estimates falling below 1,000 surgical interventions per 100,000 live births in urban areas reflect a real deficit in access to lifesaving essential obstetric surgery.^4^ Surgical care is needed not only for obstetrics and violence-related trauma but also for general morbidities in conflict zones, as demonstrated by a Médecins Sans Frontières study in the DRC province of North Kivu.^5^ Of the 3,000 operations performed over a 2-year period in that study, only one-quarter were directly related to violence, suggesting that the need for general surgical interventions in the country is so acute that when surgical services are offered in the context of emergency humanitarian aid, the uptake for general surgery is actually greater than for trauma surgery.

DRC has a population of over 60 million, with health indicators trailing behind most other countries in the region,^6^^,^^7^ mainly because of the deteriorated state of the health system and the continuous presence of armed conflict since the late 1990s. DRC's war was officially over in 2002, yet the devastating effects linger, reinforced by sporadic violence.^8^ The need for general surgical and other medical services is widespread throughout the country.

In 2006, in an attempt to address these prevalent public health problems, the DRC Ministry of Health (MOH) expressed its commitment to the principles of primary health care with the creation of its *Stratégie du Renforcement du Système de Santé* (Health Systems Strengthening Strategy, or HSSS), which introduced a framework within which to provide primary health care services, including basic surgical and obstetric care, at the district level (called *zone de santé*).^9^

In this article, we assess the capacity of 12 district hospitals in the DRC to provide surgical services, using the World Health Organization's (WHO's) Emergency and Essential Surgical Care (EESC) Situation Analysis Tool. We also used another WHO tool, the integrated Healthcare Technology Package (iHTP) resource planning and costing tool, to perform a theoretical costing exercise for surgical and other health services using a Congolese norms-based district hospital (i.e., a district hospital that would provide all services according to national norms). Our hypothesis is that surgical services are affordable and represent a minor portion of a hospital's total operating budget.

## METHODS

To operationalize the HSSS objectives, the Ministry of Health developed a comprehensive resource planning database in 2007–2008. This database detailed all resources and associated budget implications necessary to provide the minimum acceptable number and range of interventions to be included in a locally appropriate district health care package. The spectrum of health care provisions at the district level is referred to as the *Paquet Complémentaire d'Activités* (Complementary Package of Activities, or CPA, i.e., complementary to the Minimum Package of Activities that is offered at health center level). The CPA is a list of services that a district hospital should provide for a normative district population of 100,000. The CPA, in turn, created the foundation to generate a hypothetical hospital, referred to as the Model Normative Hospital, for DRC's MOH and for the purposes of this study. (The MOH also details the resources necessary for the minimum package of activities, but that is beyond the scope of this paper.)

The development of the Model Normative Hospital allows a dynamic understanding of what is a reasonable, appropriate, and attainable standard of health care in the DRC. In light of this standard, we conducted a situation analysis of the availability of emergency and essential surgical care in a sample of 12 existing district hospitals (Table 1). In addition, we modeled resource and budget data for 2 of the 12 hospitals in order to complement and validate the information from the situation analysis on resource needs and budget gaps in the DRC.

**Table 1.** Hospitals Included in the Situational Analysis and Their Locations, Democratic Republic of the Congo

**Facility**	**Province**	**Region**	**District**	**Type of Hospital**
Abuzi	Equateur	North	Abuzi	District
Bwamanda	Equateur	North	Bwamanda	District
Demba	Kasaï Occidental	Central	Demba	District
Goma	North Kivu	East	Goma	Tertiary level
Kabare	South Kivu	East	Kabare	District
Kinkanda	Bas-Congo	West	Matadi	District
Kirotshe	North Kivu	East	Kirotshe	District
Lubumbashi	Katanga	South	Lubumbashi	Tertiary level
Mulongo	Katanga	South	Mulongo	NGO/Mission
Panzi	South Kivu	East	Ibanda	NGO/Mission
Wapinda	Equateur	North	Wapinda	District
Yakoma	Equateur	North	Yakoma	District

### WHO EESC Situational Analysis Tool

The WHO “Tool for Situational Analysis to Assess Emergency and Essential Surgical Care” is a questionnaire that includes 108 data points, created to analyze the availability of emergency anesthesia and surgical services in health care facilities.^10^ It was developed by the Global Initiative for EESC research group, with input from various MOHs, WHO country and regional offices, and health care providers representing all WHO regions. The tool has already been used in several developing countries to confirm enormous deficiencies in their capacity to deliver essential surgery and anesthesia care.^3^

The situational analysis tool is comprised of 4 sections assessing:

1. **Infrastructure:** availability of basic needs such as water, electricity, oxygen, operating rooms, and functioning anesthesia machines, among others
2. **Human resources:** numbers of health care providers at the facility, focusing on those who perform surgery, anesthesia, and obstetrics
3. **Equipment:** availability of items on the WHO generic list of “Essential Emergency Equipment”
4. **Interventions:** surgical procedures provided versus referred and, if referred, whether due to lack of skills, supplies, or functional equipment

The WHO Tool for Situational Analysis to Assess Emergency and Essential Surgical Care analyzes facility capacity in infrastructure, human resources, equipment, and surgical interventions.

We collected the situation analysis data at 12 district hospitals through onsite visits and interviews, in collaboration with the WHO Country Office, the DRC MOH, and local health managers and providers.

### Integrated Healthcare Technology Package Tool

The iHTP tool is an intervention-based tool for resource planning analysis, created with detailed input data reflecting specific local health care needs.^11^ The tool links health care resources to disease profiles, patient demographics, clinical practice pathways, and technology requirements, providing resource needs and budget assessment for a given country.

Following the iHTP tool methodology, we divided and subdivided each health care intervention detailed in the CPA into specific building blocks that take into account all the essential components needed to perform the intervention (i.e., human resources, medical devices, consumable items, pharmaceuticals, and health facilities infrastructure, with corresponding resource prices and time use). All these components are connected in an algorithm format within the iHTP software tool. The algorithms are dynamic pathways of the various interventions. These can be used for simulation and analysis and tailored to represent specific disease treatments, such as those created by the WHO *Surgical Care at the District Hospital* manual,^12^ as well as preventive services and management. More information on the iHTP tool and the specific data entry process in the DRC has been described in a previous paper.^13^

Salaries and prices for each essential component were entered into the iHTP software tool in 2008 USD, and an operating budget was calculated, assuming that the basic capital equipment and the building exist and are functioning. Thus, only operating costs were calculated; capital investment costs were not included in this study (Table 2).

**Table 2.** Costs Included and Not Included in the DRC Hospital Resource Planning Exercise

**Costs**	**Examples**
**Included**	
Clinical interventions	• General anesthesia
	• Applying a cast
	• X-ray
Referral costs as defined by country	• Ambulance
	• Medical services during transport
Health facility administration	• Hospital manager salary
	• Office equipment
District health administration	• District health manager salary
	• Supervisory visits
**Not Included**	
Capital investments to rehabilitate health facilities	• Building repairs or construction of new building
Preservice human resource training	• University education for medical officers
Infrastructure outside the health care facility	• Paved roads for better access

Abbreviation: DRC, Democratic Republic of the Congo.

Large medical equipment depreciation was calculated according to the actual duration of time it was used per procedure across a defined equipment-specific life cycle (usually 5–20 years). Health facility depreciation was also calculated according to usage per procedure across a defined facility building cost. Building maintenance and utilities were added to the total health facility operating budget. Consumable items and drug quantities were calculated according to the total amount necessary for each procedure. Human resource salaries were taken from the Mbudi agreement between the Congolese government and labor unions on civil servant salaries.^14^

### Establishing the DRC Benchmark: The Model Normative District Hospital

The Model Normative Hospital was designed to understand the specific needs of the Congolese population while keeping the limitations of health care delivery in the DRC at the forefront in its design. Based on the HSSS to represent a standard from the MOH, the Model Normative Hospital aims to provide essential primary health care services for all inhabitants. The hospital serves as a theoretical model for fully functioning DRC district hospitals to strive for in terms of functionality, resource usage, utilization, and interventions provided.

The main assumptions are that the hospital covers the CPA as described above for a normative district population of 100,000. The CPA includes medical, surgical, anesthetic, pediatric, and obstetrical interventions, as well as preventive and management services. Model Normative Hospital staff salaries were based on a national directive,^14^ and medical devices, equipment, drugs, and infrastructure data were based on national clinical guidelines, textbooks used in local medical schools, and expert consensus opinion.

The Model Normative Hospital utilization rate of 0.15 cases per inhabitant per year was agreed upon within the MOH after surveying utilization rates across several well-run district hospitals in the DRC. Very few hospitals were able to demonstrate utilization rates above 0.2; hence, it was decided that a realistic utilization rate, even if not high, was necessary for planning purposes in order to stay within the Congolese context, even within the realm of the Model Normative Hospital.

Facilities in the DRC were assessed for 32 EESC situation analysis interventions, ranging from simple suturing to higher skilled laparotomy. These corresponded with the CPA interventions in the Model Normative Hospital, permitting correlation to surgical budget estimates in the iHTP tool.

### Selection of Hospital Sites for Costing Data

Direct observation of patient care in 2 hospitals, Demba and Kabare Hospitals, was performed over a period of 1 month in 2008–2009. Demba Hospital is situated in Kasaï Occidentale province and Kabare Hospital in the Eastern Sud-Kivu province. These 2 hospitals were a convenience sample, selected as part of the MOH's efforts to harmonize health services across districts. Demba and Kabare districts were both supported by an NGO that expressed a need to study the costs of providing CPA services. The data and study led to the development of Demba and Kabare Hospital-specific algorithms using the iHTP software tool. The algorithms selected were based on the CPA. Hospital registries, price lists, procurement bills, and all other relevant materials were studied and discussed with the local District Management Team. Salaries were calculated by adding the government base salary and the local salary supplement paid by the NGO supporting the hospital. All data were entered into the iHTP software for resource and budget calculations. An overview of price data sources is given in Table 3.

**Table 3.** Source of Price Data in the DRC Resource Planning Exercise by Resource Type

**Resource Type**	**Source of Price Data**
Human	• Mbudi agreement + NGO salary top-up (Demba and Kabare)
Pharmaceuticals	• Price list from 3 medicine distribution centers^a^ + IDA list price
Medical equipment	• MOH and donors (per unit purchase price)
Health facility infrastructure	• Building: Ministry of Infrastructure, Public Works, and Reconstruction (per m^2^)
	• Cleaning: 2.5% of building per year
	• Utility management: 1.5% of building per month
	• Maintenance: 2.5% of building per year
Consumable/disposable items	• MOH and district hospitals (per unit purchase price)

Abbreviations: DRC, Democratic Republic of the Congo; IDA, International Development Assistance; MOH, Ministry of Health; NGO, nongovernmental organization.

a Association Régionale D'Approvisionnement en Médicaments Essentiels (ASRAMES), Centrale de Distribution de Médicaments Essentiels du Kasaï Occidental (CADIMEK), and La Centrale d'Achat et de Distribution de Médicaments Essentiels de Kinshasa (CAMESKIN).

## RESULTS

We first present an analysis of the operating budget and utilization of surgical services for the Model Normative Hospital, Demba Hospital, and Kabare Hospital. Then we provide data from the situation analysis of surgical (including anesthesia and obstetrics) care in 12 district hospitals, including Demba and Kabare Hospitals.

### iHTP Results

The total operating budget necessary for *all* hospital services at the Model Normative Hospital was calculated at US$11.86 per inhabitant per year at a utilization rate of 0.15 cases per inhabitant per year (Table 4). This total comprises all necessary health care resources, including human resources, consumable supplies, equipment, drugs, and infrastructure (including utilities), for a CPA that includes curative medical, obstetric, pediatric, surgical, and anesthesia services as well as preventive services and management activities. We calculated the operating budget for surgical services specifically (interventions ranging from simple wound suturing to obstetric and anesthesia interventions) at US$2.17 per inhabitant per year, representing 18% of the total operating budget for all hospital services and 33.3% of the total patient caseload.

**Table 4.** Calculated Operating Budget and Utilization Rate^a^ for Selected DRC Hospitals by Type of Hospital Service

**Hospital**	**Surgery (including obstetrics)**	**Total (for all hospital services)**	**Surgery Component of Total**
**Normative Hospital**			
Costs	$2.17	$11.86	18.30%
Utilization rate	0.05	0.15	33.30%
**Demba Hospital**			
Costs	$0.08	$0.31	24.20%
Utilization rate	0.003	0.03	9.00%
**Kabare Hospital**			
Costs	$0.69	$3.40	20.20%
Utilization rate	0.01	0.15	3.33%

Abbreviation: DRC, Democratic Republic of the Congo.

a Costs are per inhabitant per year in US$. Utilization rate is the number of cases per inhabitant per year.

The operating budget for surgical services at the Model Normative Hospital was US$2.17/inhabitant/year, or 18% of the hospital's total operating budget.

At Demba Hospital, the operating budget required for surgical interventions, at US$0.08 per inhabitant per year, comprised 24% of the total operating budget necessary for all hospital services. The proportion of the patient caseload requiring surgery was found to be 9% of the total caseload.

At Kabare Hospital, the operating budget necessary for surgical interventions, at US$0.69 per inhabitant per year, comprised 20% of the total operating budget necessary for all hospital services. Only 3.33% of the total patient caseload represented surgical interventions.

For a detailed analysis of the cost of surgical services at Demba and Kabare Hospitals, see the supplementary material.

### Situational Analysis Results

We completed the situational analysis tool for 12 district hospitals, each serving as the first referral level for a collection of health centers in a specific health district. The populations served at the district level ranged from 70,000 to 252,000, with notable differences in the capacities of individual facilities as detailed below.

The basic infrastructure varied between facilities (Table 5). The average number of beds was 150, with a range of 65 to 350. Hospital admissions ranged from 600 yearly to 5,000. Five hospitals had only 1 operating room, while 3 other hospitals had 4 or more operating rooms. Only 3 hospitals had uninterrupted running water and electricity. Four hospitals were without running water and 5 had interrupted running water. Five facilities had uninterrupted electricity, 5 others had interrupted electricity, and the remaining 2 did not have electricity at all.

**Table 5.** Basic Infrastructure Capacity of Selected DRC District Hospitals

**Equipment/Supplies**	**Abuzi**	**Bwamanda**	**Demba**	**Goma**	**Kabare**	**Kinkanda**	**Kirotshe**	**Lubumbashi**	**Mulongo**	**Panzi**	**Wapinda**	**Yakoma**
Catchment area	80,000	200,000	252,000	100,000	155,000	165,000	280,000	200,000	100,000	150,000	70,000	100,000
No. of beds	65	242	65	245	150	150	150	200	150	350	90	65
No. of admissions/year	850	5,000	1,500	5,000	5,000	4,000	5,000	4,032	3,500	5,000	600	1,500
No. of surgical cases/year	250	3,500	600	1,000	1,500	850	850	2,345	850	3,500	150	250
No. of operating rooms	1	2	1	2	4	4	1	3	3	5	1	1
Running water, frequency of availability	X	O	X	✓	O	✓	O	O	O	✓	X	X
Electricity, frequency of availability	O	✓	O	✓	O	✓	✓	O	X	✓	X	O

Abbreviations: DRC, Democratic Republic of the Congo; ✓, Always; X, Never; O, Sometimes.

Of the 12 hospitals, only 2 (Bwamanda and Panzi) were fully equipped with basic essential equipment and supplies (Table 6). In addition, only 4 facilities had a functioning anesthesia machine and could provide general anesthesia (data not shown). Six facilities were without an oxygen supply. Similarly, 8 hospitals lacked consistent access to mask and tubing to connect to an oxygen source. Three facilities did not have a resuscitator bag valve and mask, 5 facilities did not have an oropharyngeal airway, and 6 facilities did not have chest tube insertion equipment.

**Table 6.** Frequency of Availability of Essential Equipment and Supplies at Selected DRC Hospitals

**Equipment/Supplies**	**Abuzi**	**Bwamanda**	**Demba**	**Goma**	**Kabare**	**Kinkanda**	**Kirotshe**	**Lubumbashi**	**Mulongo**	**Panzi**	**Wapinda**	**Yakoma**
Gloves, sterile, sizes 6–8	O	✓	✓	✓	✓	✓	✓	✓	✓	✓	O	✓
Oropharyngeal airway, adult	X	✓	X	✓	✓	✓	X	O	✓	✓	X	X
Resuscitator bag valve and mask, adult	X	✓	X	O	✓	O	✓	✓	✓	✓	X	O
Mask and tubing to connect oxygen supply	X	✓	X	✓	X	O	✓	O	O	✓	X	X
Oxygen source (cylinder/concentrator)	X	✓	X	✓	X	O	✓	✓	X	✓	X	X
Chest tubes insertion equipment	X	✓	X	✓	X	✓	X	O	O	✓	X	X

Abbreviations: DRC, Democratic Republic of the Congo; ✓, Always; X, Never; O, Sometimes.

From the 32 interventions surveyed on the situational analysis tool, only 2 hospitals, Panzi and Lubumbashi, were found to provide all the essential services (Table 7). The deficits in essential procedures varied from no deficits to 17, with an average of 7 essential procedures unavailable across this sample. For general and pediatric surgery, 7 of 12 facilities had deficiencies.

**Table 7.** Range of Surgical Interventions Performed at Selected DRC Hospitals

**Procedure**	**Abuzi**	**Bwamanda**	**Demba**	**Goma**	**Kabare**	**Kinkanda**	**Kirotshe**	**Lubumbashi**	**Mulongo**	**Panzi**	**Wapinda**	**Yakoma**
**General and Pediatric Surgery**												
Resuscitation	+	+	+	+	+	+	+	+	+	+	−	−
Abscess incision and drainage	+	+	+	+	+	+	+	+	+	+	+	+
Biopsy	−	+	−	−	+	+	−	+	+	+	−	−
Hydrocele	+	+	+	+	+	+	+	+	+	+	+	+
Hernia	+	+	+	+	+	+	+	+	+	+	+	+
Appendectomy	+	+	+	+	+	+	+	+	+	+	+	+
Laparotomy	+	+	+	+	+	+	+	+	+	+	+	+
Contracture release/skin graft	−	+	−	+	+	+	−	+	+	+	−	−
Tracheostomy/Cricothyroidotomy	−	+	−	+	−	+	+	+	+	+	−	−
Congenital hernia	+	+	+	+	+	+	+	+	+	+	+	−
Neonatal surgery	−	+	−	+	−	+	+	+	+	+	+	+
Male circumcision	+	+	+	+	+	+	+	+	+	+	+	+
**Orthopedics and Trauma**												
Suturing	+	+	+	+	+	+	+	+	+	+	+	+
Wound debridement	+	+	+	+	+	+	+	+	+	+	+	+
Burn	+	+	+	+	+	+	+	+	+	+	−	−
Foreign body removal	−	+	−	+	+	+	+	+	+	+	−	−
Joint dislocation	−	+	−	+	−	−	−	+	+	+	−	−
Closed fracture repair	−	+	−	+	−	+	+	+	+	+	−	−
Open fracture repair	−	+	−	+	−	+	−	+	+	+	−	−
Osteomyelitis/septic arthritis drainage	−	+	−	+	+	+	−	+	+	+	+	−
Amputation	−	+	−	+	+	+	+	+	+	+	−	−
Chest tube insertion	−	+	+	+	−	+	−	+	+	+	−	−
**Obstetrics/Gynecology and Other**												
Dilation and curettage	+	+	+	+	+	+	+	+	+	+	+	+
Tubal ligation	+	+	−	+	+	−	+	+	+	+	−	+
Cesarean delivery	+	+	+	+	+	+	+	+	+	+	+	+
Obstetric fistula	−	+	−	−	−	+	−	+	+	+	−	−
Cystostomy	+	+	−	+	+	+	+	+	+	+	+	+
Urethral stricture dilation	−	+	+	+	+	+	+	+	+	+	−	−
**Anesthesia Care**												
Ketamine	+	+	+	+	+	+	+	+	+	+	+	+
Regional	−	+	−	+	+	+	+	+	+	+	−	−
Spinal	+	+	+	+	+	+	+	+	+	+	+	+
General anesthesia	−	−	−	+	−	+	−	+	−	+	−	−

Abbreviation: DRC, Democratic Republic of the Congo.

Only 2 of 12 hospitals provided all essential surgical services.

Six procedures in the general and pediatric surgery category were more commonly performed and were available at all facilities, consisting of abscess incision and drainage, hydrocele repair, hernia repair, appendectomy, laparotomy, and male circumcision (Table 7). For orthopedics and trauma, suturing and wound debridement were the only 2 services provided at all 12 facilities. However, treatment for dislocated joints was provided at only 5 of 12 facilities and open fracture repair at 6 of 12 facilities. Of the 10 orthopedic and trauma procedures, Yakoma was capable of offering only 2 of the procedures, Abuzi and Wapinda offered 3, and only 5 facilities offered all procedures. For obstetrics and gynecology, all facilities were able to offer cesarean deliveries and dilation and curettage. Obstetric fistula repair was available at 6 facilities. Anesthesia care was deficient at most facilities, with only 4 of 12 facilities offering general inhalation anesthesia.

The 12 facilities together lacked an average of 21% of interventions, ranging from 0% in Lubumbashi and Panzi to 56% at Yakoma (Table 8).

**Table 8.** Summary of Surgical Deficiencies at Selected DRC Hospitals Compared With Benchmark of DRC Model Normative Hospital

****	**Normative**	**Abuzi**	**Bwamanda**	**Demba**	**Goma**	**Kabare**	**Kinkanda**	**Kirotshe**	**Lubumbashi**	**Mulongo**	**Panzi**	**Wapinda**	**Yakoma**
Catchment area, thousands	100	80	200	252	100	155	165	280	1,000	100	1,000	70	100
Maximum distance to next facility, km	NA	65	2,000	90	300	65	250	15	8	–	10	65	65
EESC capacity (of total 32 interventions)	32	17	31	17	30	24	30	23	32	31	32	15	14
Surgical intervention deficiencies^a^	0%	47%	3%	47%	6%	25%	6%	28%	0%	3%	0%	53%	56%
Cost per inhabitant per year, US$	2.17	NA	NA	0.08	NA	0.69	NA	NA	NA	NA	NA	NA	NA

Abbreviations: DRC, Democratic Republic of the Congo; EESC, emergency essential and surgical care; NA, not applicable.

a Calculated as 100 – (EESC capacity/32).

Patients traveled between 35 km and 150 km to seek surgical care in the district hospitals observed in this study. If the surgical need was not met in the district hospitals observed, the farthest distance to the next facility to access surgical care ranged from 8 km to 300 km, and in one extreme case, patients from Equateur province traveled as far as 2,000 km to the capital Kinshasa to be treated (Table 8). The median distance to the next closest facility was 65 km.

## DISCUSSION

This article presents a comprehensive resource and operating budget analysis of anesthesia and surgical (including obstetrics) services within a cohort of Congolese district hospitals. As the first of its kind using a normative model, the in-depth analysis is based not on international standards but on what is considered locally appropriate. This method makes it possible to estimate the minimum operating budget for surgical services at the Model Normative Hospital, which demonstrated that the cost for surgery is proportionally low as a percentage of the total operating cost at the district hospital level in the DRC. This finding is important when comparing deficiencies between existing district hospitals and the normative model because of the resulting resource allocation implications.

Analysis of the operating budget necessary for surgical (including obstetrics and anesthesia) services at the Model Normative Hospital demonstrated that 18.3% of the total operating budget, or US$2.17 per inhabitant per year, is necessary to deliver surgical services. Hence, providing surgical services in the DRC is not out of reach, as this 18.3% of the budget implies covering one-third of the total Model Normative Hospital patient caseload. At Demba Hospital, the operating budget required for surgical interventions, at US$0.08 per inhabitant per year, comprised 24% of the total operating budget necessary for all Demba hospital services. The proportion of the patient caseload requiring surgery was found to be 9% of the total caseload. At Kabare Hospital, the operating budget necessary for surgical interventions, at US$0.69 per inhabitant per year, comprised 20% of the total operating budget necessary for all Kabare hospital services. Only 3.33% of the total patient caseload represented surgical interventions. The explanation for this inverse finding is that the proportional budget allocated to surgery is higher if the overall and surgical caseload (utilization rate) of a given hospital is low.

**Figure:**
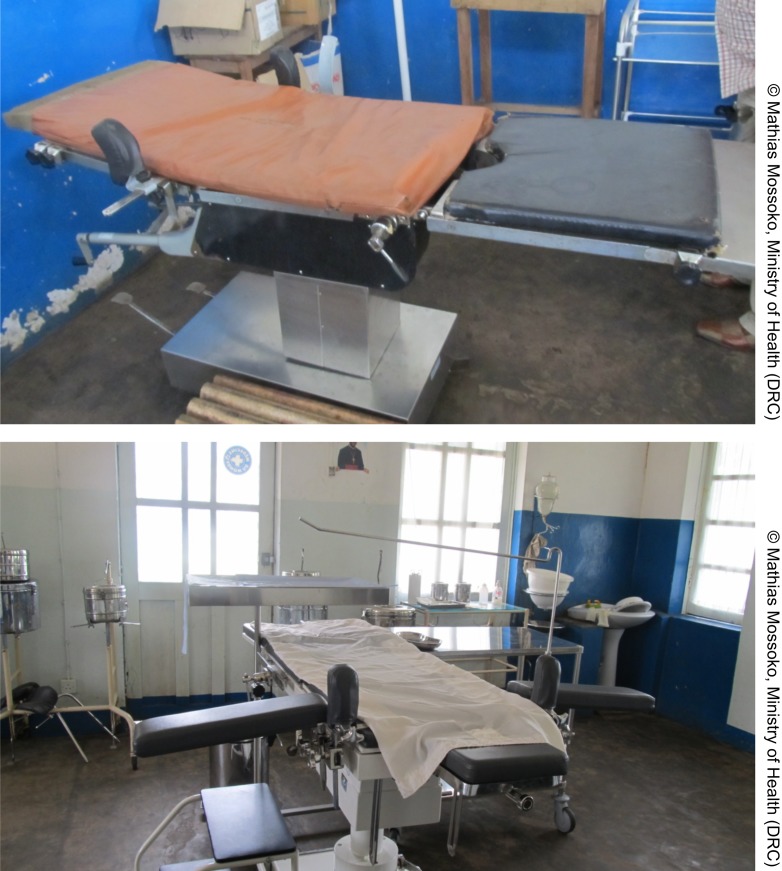
An ill-equipped operating room at a district hospital in the DRC (top) compared with a fully equipped one in another district (bottom).

As anticipated, there are remarkable shortcomings at the district hospitals within all categories of the situational analysis. From basic infrastructure such as running water and electrical power to the specific needs of surgical services, there is a glaring lack of basic supplies, personnel, and equipment. These deficiencies contribute to the limitations in availability and capacity to provide lifesaving surgical services across our sample of DRC district hospitals. An average of 21% of lifesaving interventions were absent from the facilities compared with the Model Normative Hospital.

On average, 21% of lifesaving interventions were absent from the surveyed facilities.

While the deficiencies in these hospitals are broad and pervasive, the situational analysis permits a degree of understanding of these facilities' capabilities and areas in which capacity is the weakest. Notably absent from many facilities is the capacity to provide general anesthesia with functioning anesthesia machines. Most hospitals rely on local, spinal, or IV sedation for anesthesia services without regard to complexity or length of operation. Furthermore, the provision of supplementary oxygen is essential in any first-referral facility yet only reliably available in 6 of 12 facilities we studied; it is entirely absent from 6 facilities. The need for supplementary oxygen is still largely unmet, despite the inclusion of oxygen concentrators in this study. On the other hand, all facilities provide certain general and pediatric procedures, including abscess incision and drainage, hydrocele repair, hernia repair, appendectomy, laparotomy, and male circumcision. These capabilities denote a baseline level of proficiency in surgical and anesthetic services, implying that building upon this framework to include all essential services is within reach.

In light of these challenges, deficiencies, and far-reaching effects of inadequate surgical and anesthetic care, our costing exercise with the Model Normative Hospital shows that bridging the existing surgical and anesthetic framework to meet HSSS standards is a rational and thoughtful expenditure. The surgical needs of the population can be inferred from the utilization rate of the Model Normative Hospital, which was calculated based on a survey of facility registries from well-run Congolese hospitals. At 33% of the total patient load at the Model Normative Hospital, surgical need is high. Yet looking more closely at Demba and Kabare Hospital surgical caseloads, these needs are not met. Only 9% and 3.33%, respectively, of the 2 hospitals' caseloads are surgical patients.

Reasons for the comparatively low caseloads (utilization rate) at Demba and Kabare Hospitals can be hypothesized by assessing these hospitals' infrastructure and resource base in general—patients are likely to bypass facilities within their health district due to health facility deficiencies. Both Demba and Kabare Hospitals are faced with unreliable electricity and water infrastructure as well as a total lack of an oxygen source. Both hospitals are fully or partially lacking equipment and supplies necessary for adequate surgical and anesthetic care. The situational analysis shows that Demba Hospital staff refer patients to facilities 90 km away for most orthopedic/trauma care. Similarly, Kabare Hospital refers patients with open and closed fractures and joint dislocations to facilities 65 km away. Thus, there is suboptimal use of the existing framework at these hospitals, and in the 12 facilities examined, patients traveled between 35 km and 150 km in an effort to seek surgical care within their health district, and up to 300 km (or in one extreme case, 2,000 km) to find care elsewhere. When the district hospital is insufficient, the result is unnecessary or difficult referrals to distant facilities.^15^ As in many low-income countries, transfer to another facility is often the responsibility of the patient. The issues of safety, time, income, and lost work can be very difficult for a family to bear if a hospital transfer is needed. Furthermore, the long distances are frequently prohibitive, particularly with severe medical conditions, leading to disability or death.

Because the Model Normative Hospital was developed by DRC's HSSS standards, deficiencies indicate a need for greater investment in the health sector and, more specifically, in surgical services. It can be inferred that bringing these hospitals to the level of the Model Normative Hospital will improve the health care system as a whole. At the population level, the detriment to a community when even 1 of the 32 essential procedures is absent is far-reaching beyond the implications for the individual patient. Striving to provide a full complement of services is essential because of the interconnectedness of clinical care.

### Study Limitations

Limitations of this study are the general lack of available and reliable data, especially concerning utilization rates, caseloads, and other facility-based data. Another limitation is the nature of the convenience sample of district hospitals representing the North, South, East, West, and Central regions of the DRC. Findings cannot be generalized to the entire country. The situational analysis is only a “snapshot” needs assessment of district hospitals. Also, our resource planning study assessed only the resources and associated operating budget necessary at the district hospital level; large capital investments in roads as well as basic public infrastructure such as electricity and water will require much larger sums to be provided at the national level in order for the district level to function appropriately.

## CONCLUSION

Surgery has been neglected from global health aid, but the accumulating proof of its large health impact and its proportionally low required service delivery budget are cause for a larger focus on making surgical (including obstetric) interventions available to the world's poorest communities. District (first-level referral) hospitals must be better funded to provide such services. More often than not, the level of service delivery used for surgical care (tertiary) is not appropriate in the DRC; given the low cost (i.e., <30% of overall functioning of the hospital) of providing surgical care at first-level referral hospitals, this should not be the case.

It is clear from the Demba and Kabare Hospital costing data that current spending on surgery, at least in these hospitals, is well below what is needed to achieve the DRC norm. We also know that households in the DRC contribute significantly (US$4.50/inhabitant/year^16^) to health care costs and thus directly or indirectly co-fund health services, including surgical services. Health care resource budgets must be restructured considerably in order to provide for lifesaving emergency and essential surgical care, including necessary infrastructure, human resources, equipment, pharmaceuticals, and supplies, within the primary health care package. We acknowledge the difficulty of putting in place structures and a system that would provide the necessary resources even with an adequate budget. However, these efforts to holistically strengthen the system are imperative in order to provide essential services that are not excessively expensive but will go a long way to improve population health.

## Notes

**Peer Reviewed**
